# Size Dependent Uptake and Hemolytic Effect of Zinc Oxide Nanoparticles on Erythrocytes and Biomedical Potential of ZnO-Ferulic acid Conjugates

**DOI:** 10.1038/s41598-017-04440-y

**Published:** 2017-06-23

**Authors:** E. Preedia Babu, A. Subastri, A. Suyavaran, K. Premkumar, V. Sujatha, B. Aristatile, Ghedeir M. Alshammari, V. Dharuman, C. Thirunavukkarasu

**Affiliations:** 1 Department of Biochemistry and Molecular Biology, Pondicherry University, Puducherry, 605 014 India; 2 Cancer Genetics and Nanomedicine Laboratory, Department of Biomedical Science, Bharathidasan University, Tiruchirappalli, 620 024 India; 3 Department of Chemistry, Periyar University, Salem, 636011 India; 4 Department of Food Science and Nutrition, College of Food and Agricultural Science, King Saud University, P.O. Box 2460, Riyadh, 11451 Saudi Arabia; 5 Molecular Electronics Laboratory, Department of Bioelectronics and Biosensors, School of Life Sciences, Alagappa University, Karaikudi, 630 003 India

## Abstract

Despite zinc oxide nanoparticles (ZnONPs) being increasingly used as carriers in biomedical fields due to their multifaceted properties and therapeutic importance, better understanding of the mechanisms and cellular consequences resulting from their interaction with cells and cellular components has been warranted. In the present study, we investigate the size-dependent interaction of ZnONPs on RBCs, and its impact on cell viability, DNA damage, ROS generation and morphological changes, employing cellular and analytical methods. Size, charge, stability and solubility were confirmed by DLS, zeta potential, ICP-AES and TEM analysis. Further ICP-AES, TEM, spectroscopic observations and cell based assays showed that ZnONPs exhibited a size dependent impact on RBCs and haemoglobin (Hb), particularly size <50 nm. Conversely, ferulic acid (FA) conjugates and serum albumin significantly reduced the adverse effects exhibited by ZnONPs. The extent of DNA damage and ROS generation is comparatively low in ZnONPs-FA than in ZnONPs alone treated cells. Thus our study documents a novel conceptualization delineating the influence of size on the material properties and therapeutic potential of nanoparticle.

## Introduction

Metal oxide nanoparticles have gained great scientific interest because of its multifaceted properties and therapeutic importance^1–3^. Among the metal oxide nanoparticles zinc oxide nanoparticles (ZnONPs), being increasingly used in biomedical fields due to its unique chemical and physical properties. Numerous studies demonstrate that it can activate ROS-mediated cytotoxic pathway and apoptosis^4–7^. Interestingly, ZnONPs have been used as carrier for various chemotherapeutic drugs such as cisplatin, doxorubicin and 5-flurouracil^8, 9^. Though, ZnONPs have pervasive application in various medical fields, its toxicity has been highest concern and important focus. Although, acute and chronic neurotoxic, genotoxic effects of ZnONPs were demonstrated in time and concentration-dependent manner using *in vitro* and *in vivo* models^10, 11^, biocompatibility and intrinsic hemolytic activity of ZnONPs remains to be elucidated.

Chemotherapeutic drugs delivered through intravenous route interact with major components of the circulatory system^12^. As red blood cells (RBCs) being most important cells in circulatory system, interaction of the drug with RBCs damages its membrane and interacts with hemoglobin (Hb). Interaction of xenobiotics with Hb alter its structure and cause conformational changes, thus decreasing its oxygen binding capacity and increasing pro-oxidative effect^13^. Some of the chemotherapeutic drugs including platinum based drugs and tamoxifen induce hemolytic anemia and interact with Hb, changing its conformation, thus limiting their utilization in cancer treatment^14, 15^. Similarly nanoparticles size, stability, shape, surface characteristics, route of exposure and medium are some of the key factors conferring their utilization and/or exerting toxicity^16, 17^. Hence, critical analysis on size-dependent effect on RBCs and Hb becomes most essential part in understanding and evaluating the adverse effects of ZnONPs.

In order to reduce the hemolytic activity and improve the efficacy of chemotherapeutic drugs, polymer based drug delivery carriers and co-administration of drugs with phytochemicals have been practised^18^. Many phytochemicals demonstrated their efficacy either alone or in combination with chemo-drugs. Curcumin, genistein and quercetin are used as adjuvants to potentiate the chemotherapeutic efficacy of drugs such as cisplatin, 5-flurouracil and doxorubicin^19^. Ferulic acid (FA) is a hydrocynnamic acid, well known for its antioxidant, anti-aging, anti-diabetic, anti-inflammatory actions^20^. Recent studies proved its protective effect on Hb from glycation and known for its photo-, hepato- and neuroprotective role^21^. Furthermore, FA plays a crucial role in inducing differentiation and apoptosis by inhibiting anti-apoptotic factors, and thus inhibits proliferation and metastasis of cancer cells^22^. Recently we have shown the reducing potential of FA and have been utilized for the synthesis of ZnONPs from zinc acetate^23^. In view of evaluating the biomedical potential of ZnONPs, the present study was initiated to critically evaluate the size-dependent hemolytic behavior and interaction with Hb, using chicken RBCs.

## Results

### Size dependent separation of ZnONPs

We procured the commercially available ZnONPs and characteristic sizes of those nanoparticles were confirmed by DLS and were ranging from 0–307 nm. Nanoparticles were subsequently separated into different sizes using rate zonal density gradient centrifugation. Further the size distribution was analyzed by DLS (Figure S1A) showing ZnONPs bands in 25%, 50% and 75% sucrose gradient and the size distribution ranges from <50 nm, 50–100 nm and >100 nm (Figure S1B–E) respectively. Further the different size was confirmed by transmission electron microscopy (Figure S1F–I).

### Stability and solubility of ZnONPs

The stability of various size ZnONPs in different media such as water, FA, saline and PBS was studied by DLS at 1 h and 24 h on different storage conditions (−80 °C, 4 °C and RT). The results shown in Table 1 indicate that ZnONPs size <50 nm and 50–100 nm were stable in water at all storage conditions. On the other hand particle size >100 nm was not stable at 24 h. Presence of FA reveals that it can stabilize the lower size (<50 nm and 50–100 nm) ZnONPs. Anti-aggregation property of ZnONPs may be due to the surface stabilizing property of the functional group(s) of FA. In addition, result shows that ZnONPs was more stable at −80 °C compared to 4 °C and RT. Further we found that, ZnONPs particles aggregate within 1 h in saline and PBS. Repeated freezing and thawing showed negative impact on the size of ZnONPs with respect to their dispersity. Since ZnONPs (<50 nm) showed stability difference in the different medium, we analyzed the zeta potential. Zeta potential was found to be −4.21, −4.1, −1.5 and +41.6 respectively in water, PBs, saline and FA medium. It is known that colloidal solution with Zeta potential (ξ) ± 30 mV is stable and ξ ± 40 mV indicate good stability. Thus ZnONPs in FA medium has good stability. Zeta potential was found to be similar in water, PBS and Saline indicating less stability. However, DLS data shows that ZnONPs is stable in water and FA medium. From the above experiment, we intended to check the solubility of ZnONPs in water at different static condition by monitoring the ionization of ZnONPs using ICP-AES. The result showed (Table S1) that there was no significant difference in the ionization of ZnONPs at different static condition but after 24 h a slight increase was noticed, furthermore the ionization of ZnONPs was significantly lesser than ZnCl_2._ These experiments indicate that ZnONPs is stable in water and FA medium.

**Table 1:** The Stability of ZnONPs (<50, 50–100 and >100 nm) dispersed in water, PBS, saline and FA (50 µM) solution was scrutinized at different temperature (RT, 4 °C, −80 °C) by monitoring the average size distribution using DLS analysis at 1 h and 24 h.

Particulars		1 h				24 h			
		Water	PBS	Saline	FA	Water	PBS	Saline	FA
<50 nm	**RT**	31 ± 4	745 ± 63	849 ± 42	29 ± 2	33 ± 3	1104 ± 65	1134 ± 49	42 ± 4
	**4 °C**	29 ± 4	735 ± 64	835 ± 51	26 ± 3	31 ± 3	877 ± 52	1120 ± 62	38 ± 5
	**−80 °C**	28 ± 4	730 ± 48	820 ± 48	25 ± 4	31 ± 4	773 ± 41	1009 ± 48	38 ± 3
50–100 nm	**RT**	65 ± 5	780 ± 69	928 ± 44	53 ± 3	70 ± 4	870 ± 56	1206 ± 49	62 ± 3
	**4°C**	64 ± 3	775 ± 84	910 ± 67	55 ± 4	66 ± 5	988 ± 48	1189 ± 47	65 ± 3
	**−80 °C**	64 ± 2	760 ± 68	905 ± 47	55 ± 4	65 ± 4	783 ± 74	1170 ± 48	60 ± 4
>100 nm	**RT**	321 ± 32	896 ± 68	1100 ± 80	579 ± 64	708 ± 43	2100 ± 69	2525 ± 59	896 ± 5
	**4 °C**	320 ± 41	885 ± 7	1005 ± 47	565 ± 72	784 ± 44	1080 ± 58	2460 ± 48	844 ± 34
	**−80 °C**	320 ± 50	870 ± 75	989 ± 68	560 ± 41	593 ± 54	950 ± 41	2289 ± 49	579 ± 33

All values expressed as mean ± SD of three independent experiments.

### RBCs interaction

#### Hemolytic potential

**Figure 1:**
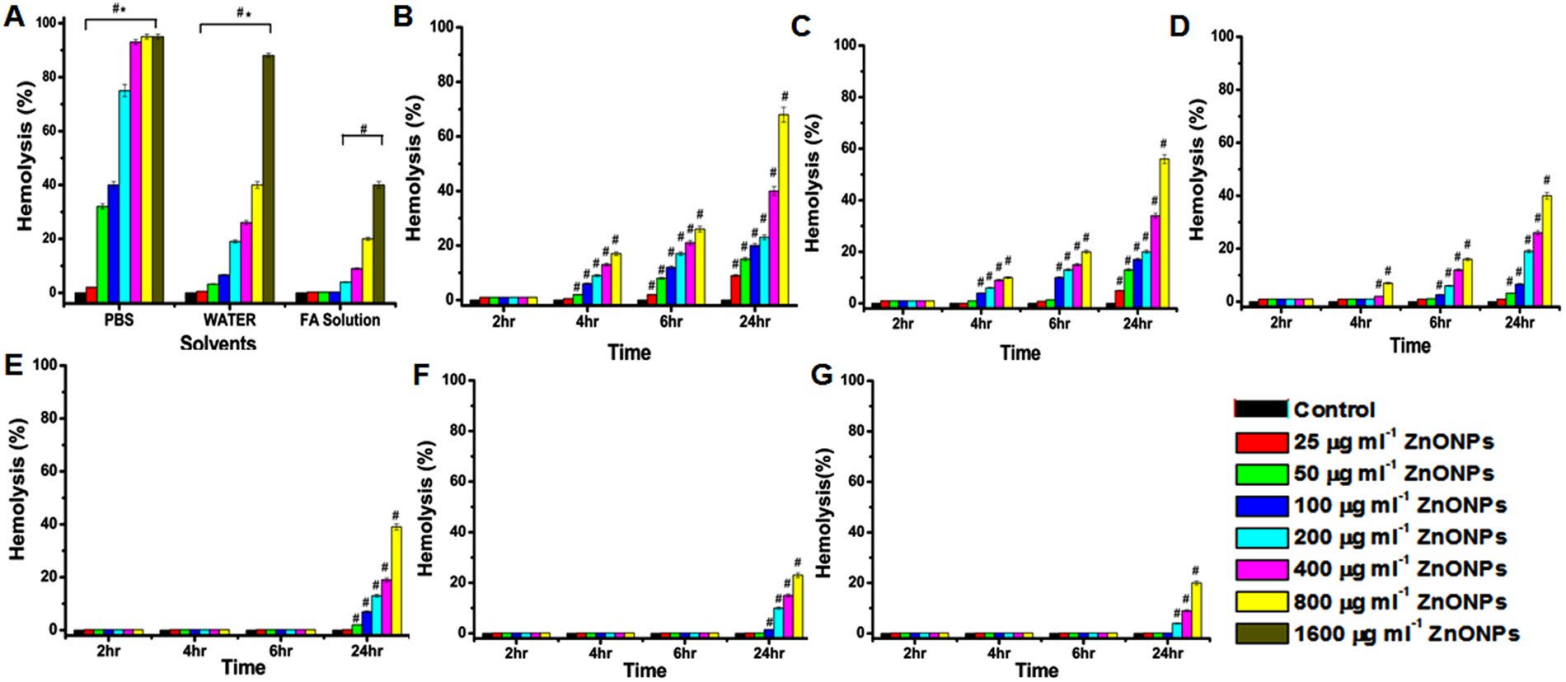
The percent hemolysis of RBCs incubated with various sizes of ZnONPs at different time and various solvent conditions. (**A**) >100 nm ZnONPs at different concentration (25, 50, 100, 200, 400, 800, 1600 µg ml^−1^) dispersed in 1x PBS, double distilled water and 50 µM FA solution for 24 h. The data represent the mean ± SD (n = 3) from three experiments. ^**#**^Represent significant difference at *P* *≤* 0.05 of PBS, water and FA solution at various concentration compared to control, *represent significant difference at *P* *≤* 0.05 of PBS and water compared to FA solution. (**B**–**D**) <50, 50–100 and >100 nm ZnONP dispersed in water for 2 h, 4 h, 6 h, 24 h (**E**–**G**) <50, 50–100 and >100 nm ZnONPs dispersed in FA for 2 h, 4 h, 6 h, 24 h. The data represent the mean ± SD (n = 3) from three experiments. ^**#**^Represents significant difference at *P* *≤* 0.05 of <50, 50–100 and >100 nm ZnONPs at various concentrations compared to control at particular temperature.

The hemolytic effect of ZnONPs in different solvents with respect to their size, concentration (25 µg ml^−1^ to 1600 µg ml^−1^) and time (2 h, 4 h, 6 h and 24 h) were analyzed. Initially we analyzed the hemolysis of ZnONPs (>100 nm) in different vehicles such as PBS, water and in the presence of FA. These results (Fig. 1A) indicate that hemolytic effect of ZnONPs decreases in order as: PBS >WATER >FA. Further we analyzed hemolytic effect of different sized (<50, 50–100 and >100 nm) ZnONPs in concentration and time-dependent manner. The result of ZnONPs dispersed in water (Fig. 1B) shows that <50 nm ZnONPs has higher hemolytic activity compared to other sizes (Fig. 1C,D). The adverse effect was increased with increasing concentration from 25 to 800 µg ml^−1^ in a time-dependent manner. The hemolytic effect of ZnONPs with FA (Fig. 1E–G) shows that significant hemolytic activity occur only at higher (>200 µg ml^−1^) concentrations at 24 h. Interestingly FA has shown its positive effect irrespective of ZnONPs size. We have screened the effect of <50 nm ZnONPs and ZnONPs with FA (24 h) on human RBCs (Figure S3) and observed similar effects.

### Morphological changes

**Figure 2:**
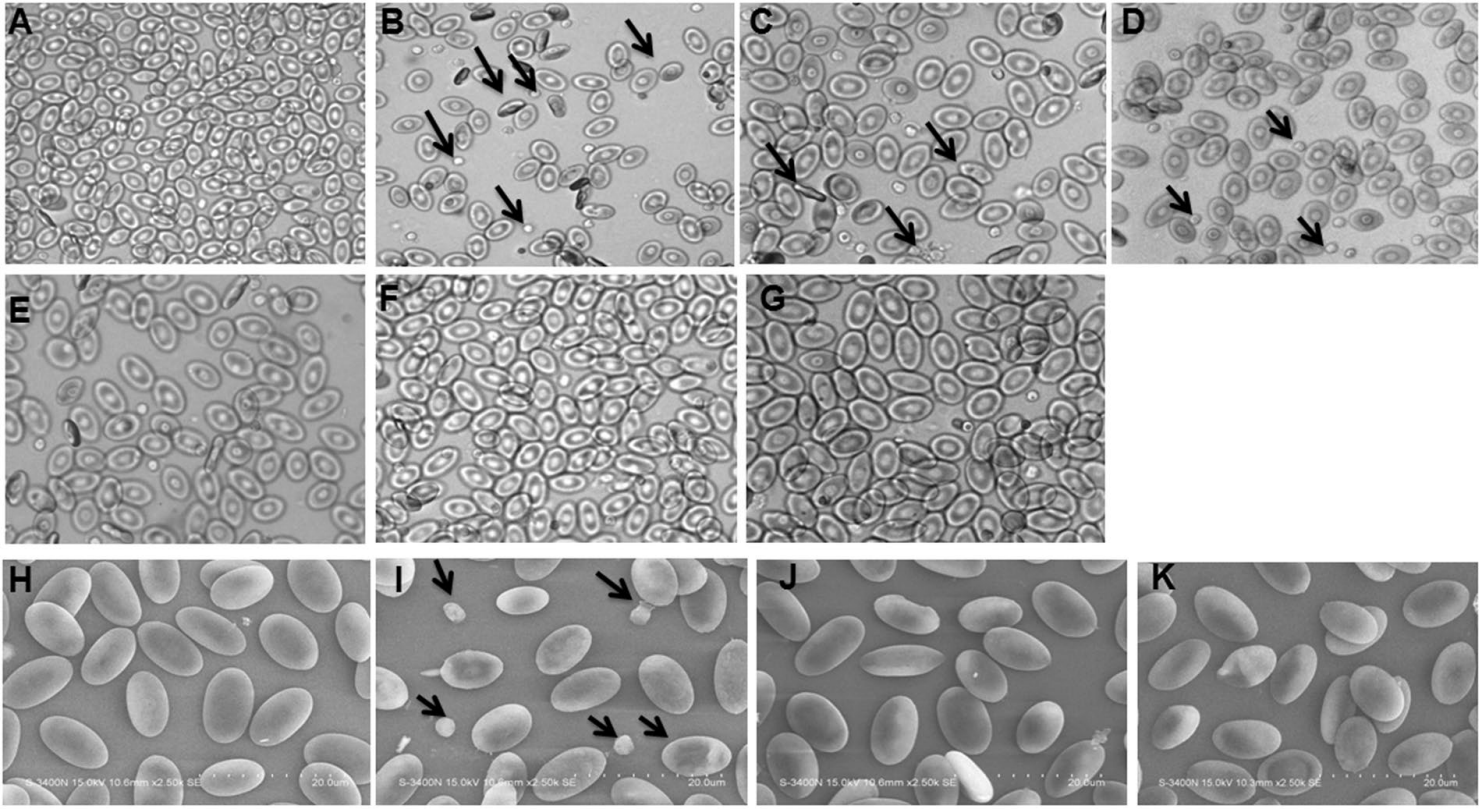
Light micrograph of RBCs alone and RBCs-ZnONPs (50 µg ml^−1^) complex at the incubation period of 24 h. (**A**) RBCs alone (**B**–**D**) RBCs treated with <50, 50–100 and >100 nm ZnONPs dispersed in water (**E**–**G**) RBCs treated with <50, 50–100 and >100 nm ZnONPs with FA (50 µM) solution.(**H**–**K**) Scanning electron micrograph of RBCs alone and RBCs treated with ZnONPs (50 µg ml^−1^) dispersed in water for 24 h.

The light microscopic images of RBCs treated with <50, 50–100 and >100 nm of ZnONPs and ZnONPs with FA, was taken after 24 h (Fig. 2). When compared with untreated RBCs (Fig. 2A) <50 nm (Fig. 2B) ZnONPs has shows membrane damage and structural deformation and the impact was decreasing with increase in size of nanoparticle (Fig. 2C,D). In the case of ZnONPs with FA the effect was significantly decreased (Fig. 2E–G). It specifies the protective role of FA on ZnONPs-induced RBCs changes. Scanning electron microscope (SEM) is used to study detailed surface topography of RBCs treated with three different sized ZnONPs, these results (Fig. 2H–K) also coincides with the light microscopic observations.

### RBCs viability

**Figure 3:**
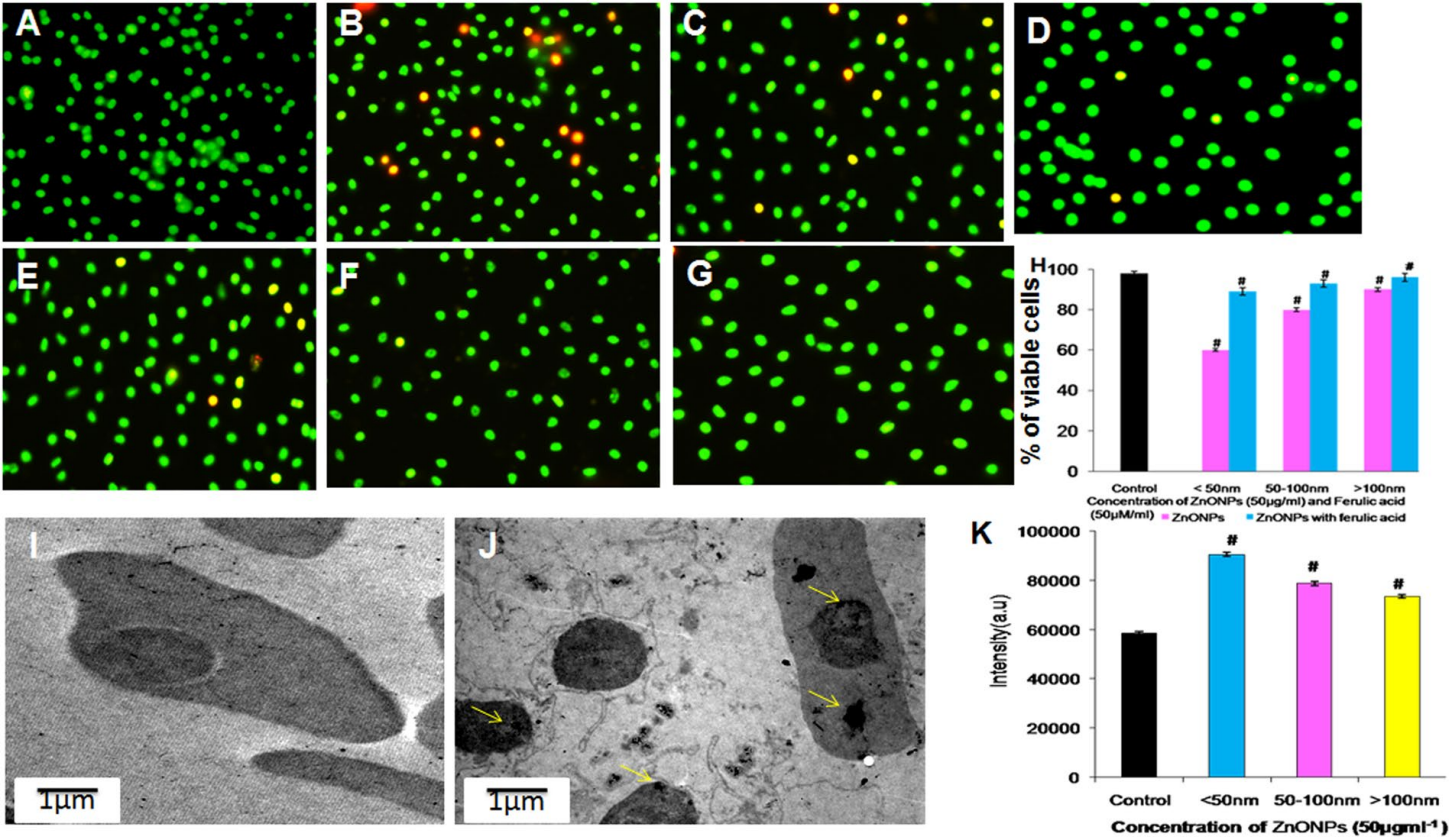
Fluorescent microscopic image of acridine orange-ethidium bromide stained RBCs and RBCs treated with ZnONPs (50 µg ml^−1^) for 24 h and visualization of ZnONPs uptake by RBCs (**A**) RBCs alone (**B**–**D**) <50, 50–100 & >100 nm ZnONPs dispersed in water (**E**–**G**) <50, 50–100 & >100 nm ZnONPs dispersed in FA (50 µM) solution. (**H**) The percentage of viable cell counted at three different fields. (**I**) Transmission electron microscopic image (TEM) of RBC cell (**J**) TEM image of RBC cells treated with <50 nm ZnONPs, yellow colour arrows indicate the internalized ZnONPs. (**K**) The internalization of ZnONPs in to RBCs was analyzed by ICP- AES. ^#^Represents significant difference at *P* ≤ 0.05 of samples compared to control.

The untreated RBCs (control) contains >98% viable cells (Fig. 3A), the percent of viable cells were comparatively less in <50 nm ZnONPs-treated sample (Fig. 3B 60%) compared to 50–100 nm (80%) and >100 nm (90%) (Fig. 3C,D). The RBCs treated with ZnONPs in the presence of FA, the non-viable cells were decreased when compared to their respective ZnONPs alone treated cells (Fig. 3E–G). The percent viable cells were shown in Fig. 3H. The results of LDH release from plasma membrane damaged cells correlated with that of cell viability assay (Figure S4).

### ZnONPs uptake study

The uptake of ZnONPs was studied by ICP- AES is a type of emission spectroscopy used for detection of trace elements in 0.5 ppm–1000 ppm levels. The internalization of <50, 50–100 and >100 nm ZnONPs by RBCs (2 × 10^6 ^cells ml^−1^) were analyzed. The emission intensity of <50 nm, 50–100 and >100 nm ZnONPs, (Fig. 3K) indicate that <50 nm ZnONPs have shown higher internalization than other sizes. TEM was used to confirm the uptake of ZnONPs. We have observed that the normal RBC shows the intact nucleus and haemoglobin (Fig. 3I) but incubation RBCs with ZnONPs (<50 nm) shows the hemolytic cells and membrane damage (Fig. 3J). Further ZnONPs was appeared as electron dense cluster in the nucleus and the cytoplasm of RBC (Fig. 3J).

### DNA damage

**Figure 4:**
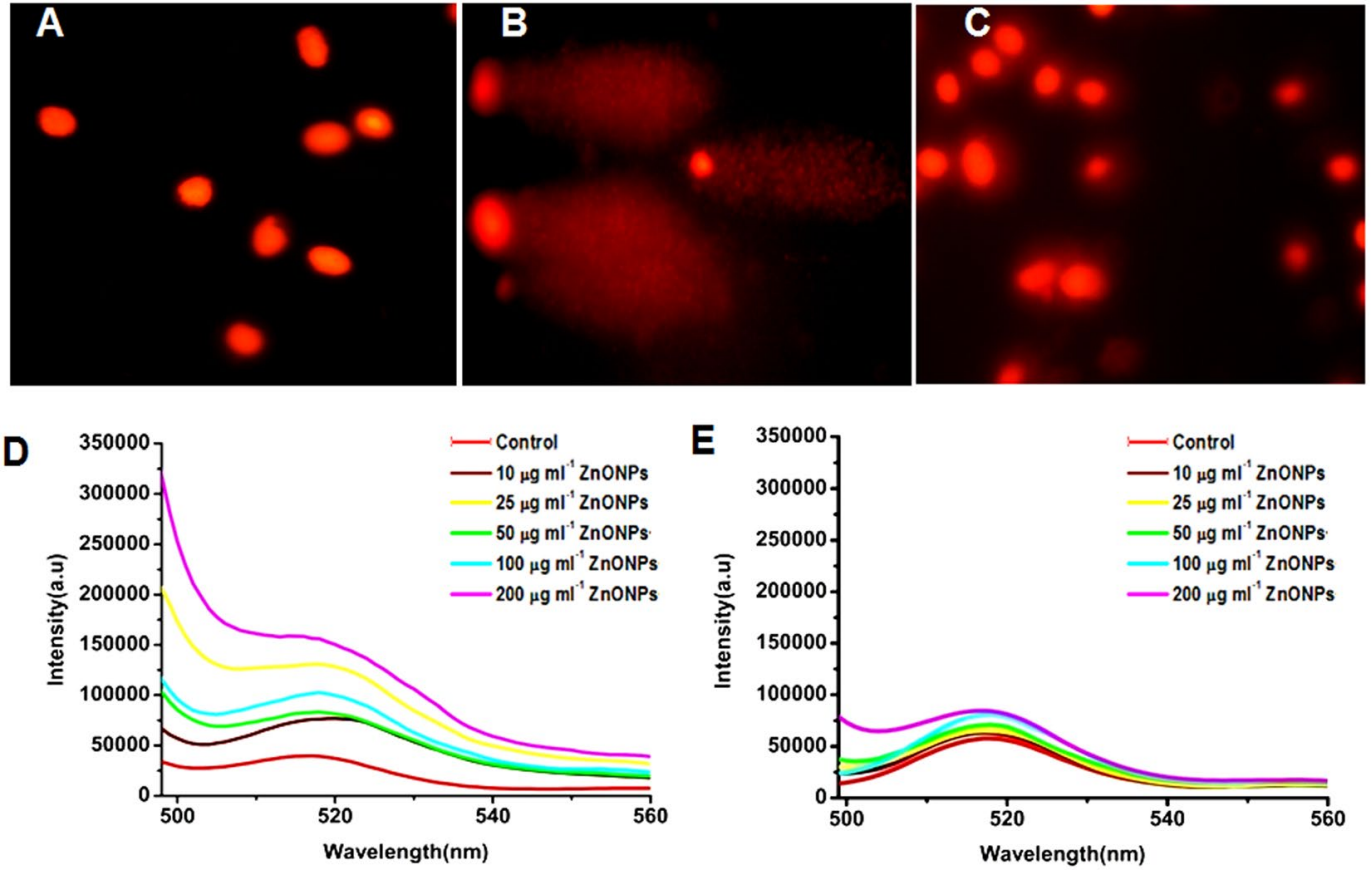
Photomicrograph of DNA damage and ROS generation. A-E: DNA damage by comet assay for monitoring DNA damage induced by ZnONPs (50 µg ml^−1^) on RBCs for 24 h. (**A**) RBCs alone (**B**) RBCs incubated with <50 nm ZnONPs dispersed in water (**C**) RBCs incubated with <50 ZnONPs with FA (50 µM) solution. (**D**,**E**) Photoluminescence spectra of ROS generated in RBCs incubated with varying concentration of ZnONPs (0, 10, 25, 50, 100, 200 µg ml^−1^) using fluorescent probe DCFDA (5 mM) (**D**) <50 nm ZnONPs dispersed in water (**E**) represent <50 nm ZnONPs with 50 µM FA solution.

The comet pattern is shown in Fig. 4 and the percent of DNA in head and tail is shown in Table S2. The results show that untreated RBCs (Fig. 4A) have higher percent of DNA in head (95%) and the tail region contains only 5% DNA. RBCs treated with <50 nm ZnONPs alone, (Fig. 4B) the percent of DNA in head region was decreased to 15.04% and that of tail region was increased to 84.96%, as compared to untreated RBCs. On the other hand, in the presence of FA the <50 nm ZnONPs treated cells have shown very less DNA damage (Fig. 4C).

### ROS generation

Formation of ROS in RBCs in the presence of <50 nm ZnONPs and ZnONPs with FA were analyzed by using DCFDA as a fluorescent probe. The different experimental condition RBCs were excited at 480 nm and the emission peak was observed at 520 nm. Comparative results shows that significant ROS generation occurs in RBCs in the presence of ZnONPs (Fig. 4D) alone and the intensity were increased with increasing concentration of ZnONPs. In the presence of FA, ROS generation was found to be decreased as compared to respective ZnONPs alone treated RBCs (Fig. 4E).

### Hemolytic effect of ZnONPs in presence of albumin and fetal bovine serum (FBS)

**Figure 5:**
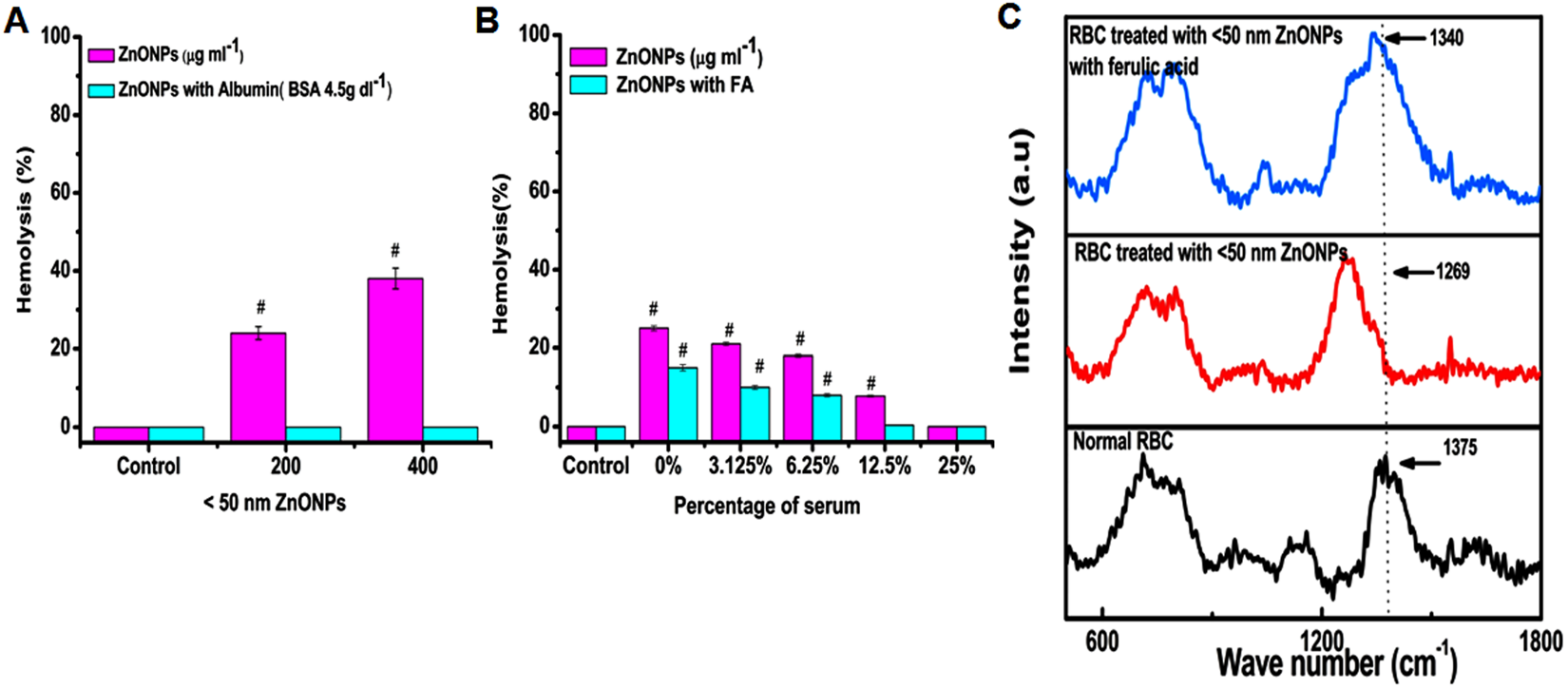
The percent of hemolysis of RBC incubated with ZnONPs in presence of albumin, various concentration of fetal bovine serum (FBS) and Raman spectra of RBCs and RBCs treated 50 µg ml^−1^ ZnONPs for 24 h (**A**) The effect of <50 nm ZnONPs (200, 400 µg ml^−1^) on the hemolysis in presence of albumin (4.5 µg dl^−1^) at 24 h. ^#^Represents significant difference at *P* ≤ 0.05 of samples compared to control. (**B**) The effect of <50 nm ZnONPs (200 µg ml^−1^) and ZnONPs with FA on hemolysis in presence of FBS (25%, 12.5%, 6.25%, 3.125%, 0%) at 24 h. ^#^Represents significant difference at *P* ≤ 0.05 of samples compared to control. (**C**) Raman specra of RBCs alone, RBCs treated with <50 nm ZnONPs dispersed in water and normal RBCs treated with <50 nm ZnONPs with FA.

Earlier studies shows that, ZnONPs interact with albumin, we assumed that interaction of ZnONPs with albumin may reduce the ZnONPs induced hemolysis in RBCs. Presence of albumin showed no hemolysis (Fig. 5A). ZnONPs incubated in the presence and absence of albumin clearly supports the above findings implying attenuating effects of albumin against ZnONPs-induced RBCs hemolysis (Figure S5). Further the hemolytic effect of ZnONPs and ZnONPs with FA was studied in different percent of serum conditions (Fig. 5B). Results show that hemolysis increases with decreasing percent of serum, on the other hand FA decreases ZnONPs induced hemolysis.

### Raman spectra

SEM analysis shown the elliptocytic RBCs, which is due the reduction in iron content in RBCs. Since Hb is the major iron binding protein in RBCs. We studied the internalized ZnONPs effect on Hb. Raman spectra showed significant peaks at four regions (Fig. 5C). The healthy RBCs has significant peak at pyrrole stretching region 1300–1400 cm^−1^, in the ZnONPs-treated samples the peak was shifted from 1373 cm^−1^ (control) to 1269 cm^−1^ (<50 nm). It indicates the C-N stretching in heterocyclic ring that surrounds the iron atom^24^ while comparing with ZnONPs in the presence of FA the effect is comparatively less which may be due to ZnONPs– FA complex decreases the affinity of ZnONPs for porphyrine ring.

### Hb interaction study

#### UV- Visble absorption

**Figure 6:**
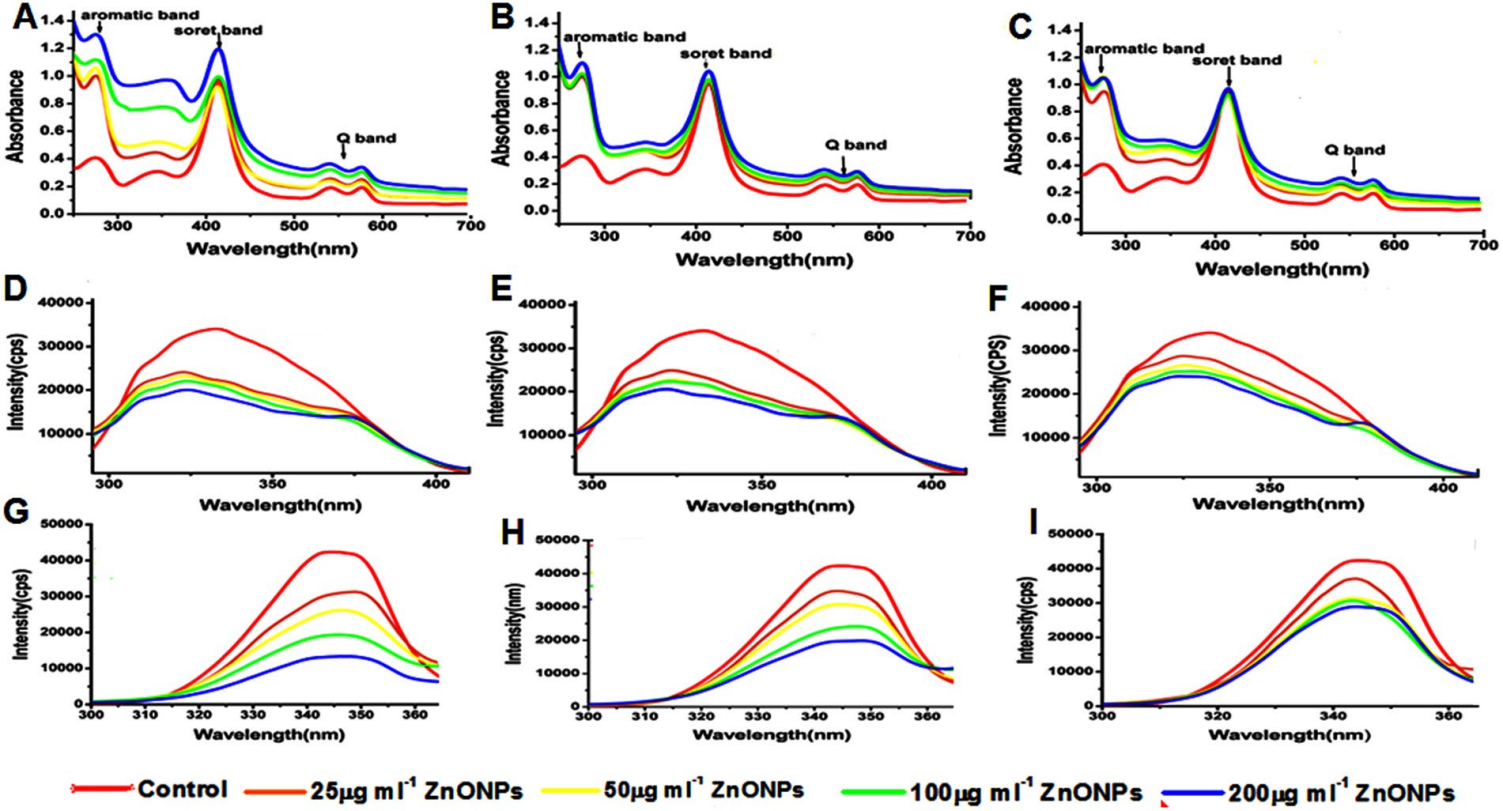
Interaction studies of hemoglobin with different sizes of ZnONPs at various concentration using spectroscopic methods (**A**–**C**) UV- visible absorption spectra of Hb and <50, 50–100, >100 nm ZnONPs (25, 50, 100, 200 µg ml^−1^) with Hb (**D**–**F**) Steady state fluorescence spectra of Hb and <50, 50–100, >100 nm ZnONPs with Hb (**G**–**I**) Synchronous fluorescence (∆λ = 60 nm) spectra of Hb and <50, 50–100, >100 nm ZnONPs with Hb.

UV- visible spectrum of Hb and Hb-ZnONPs complex is shown in Fig. 6A–C. We compared various sizes of ZnONPs, <50 nm, 50–100 nm, and >100 nm ZnONPs effect on Hb. The UV spectrum of <50 nm ZnONPs shows significant changes in Hb compared to other sizes.

### Steady state and synchronous fluorescence

Fluorescence emission spectrum of Hb and Hb with different sizes of (<50, 50–100 and >100 nm) ZnONPs indicates that the intensity of emission was decreased with blue shift while increasing concentration of ZnONPs (Fig. 6D–F), it also shows that by increasing the sizes, quenching efficacy was reduced indicating smaller size ZnONPs have higher quenching efficiency (Gopa *et al*.^25^). Stern - Volmer quenching constant (Ksv) was found to be 9.92 × 10^6^, 6.3 × 10^6^, 4.3 × 10^6^ M^−1^ (Figure S4A–C) for <50, 50–100, and >100 nm ZnONPs, these values signifies the strong quenching efficacy of ZnONPs. The bimolecular quenching constant (Kq) was found to be 3.8 × 10^15^, 2.4 × 10^15^, 1.6 × 10^15^ M^−1^ s^−1^ (Figure S6). These values are higher than diffusion controlled (10^10^ M^−1^ s^−1^) quenching process and it implies the type of quenching is static^26^.

### Synchronous fluorescence spectrum (SFS)

The SFS of Hb in the presence and absence of ZnONPs is shown in Fig. 6G–I and Figure S7. The result clearly shows that fluorescent intensity was decreased significantly with red shift in absorption maxima of tryptophan residues on binding with <50 nm ZnONPs (Fig. 6G), while increasing the size of ZnONPs 50–100 nm have slight red shift but there is no red shift >100 nm (Fig. 6H,I) moreover fluorescent intensity is also slightly decreased with increased size of ZnONPs. The same effect was observed in case of tyrosine residue, results were shown in Figure S5A–C.

### CD spectroscopy

**Figure 7:**
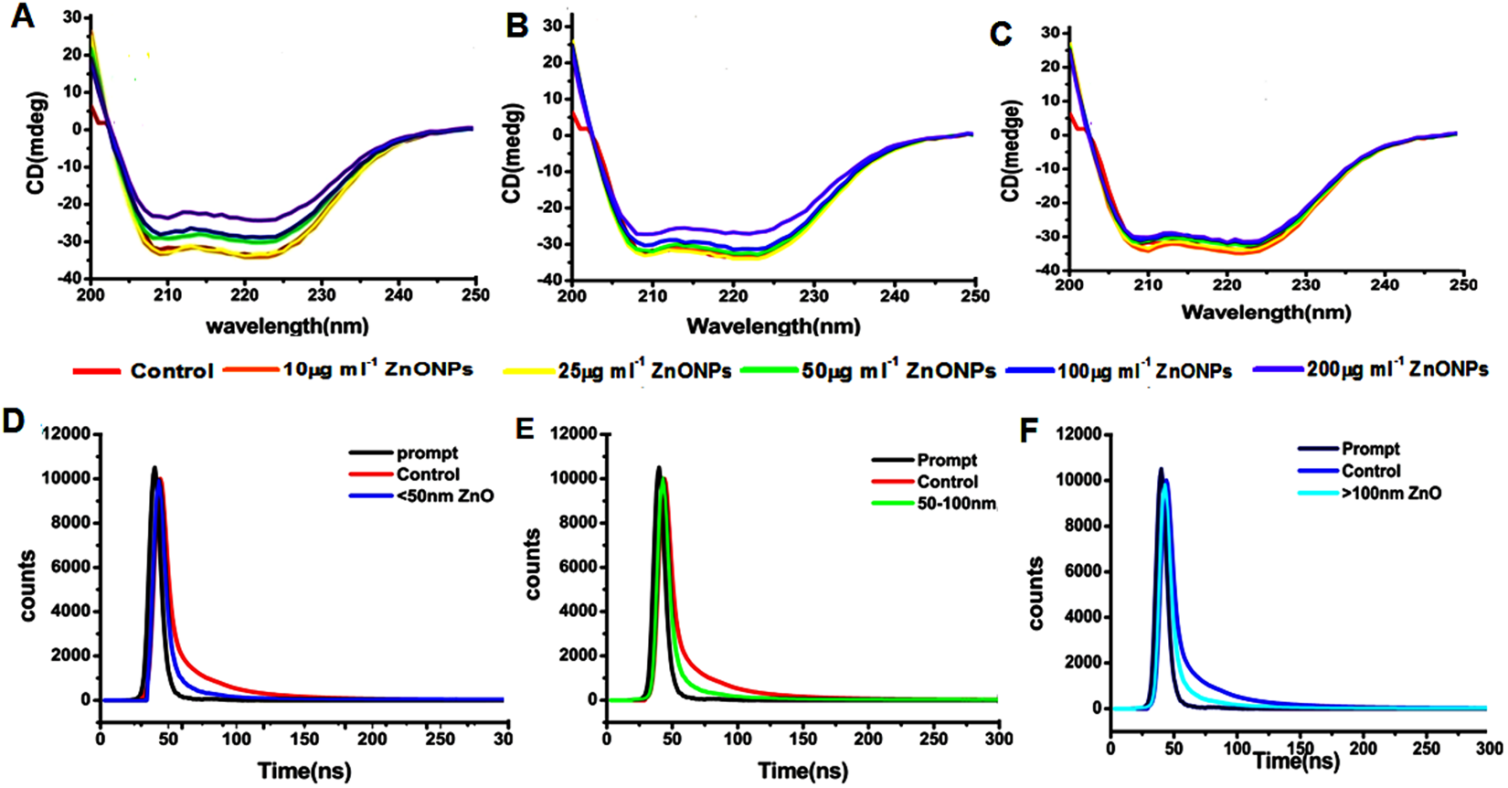
Conformational changes and lifetime decay profile of hemoglobin and hemoglobin- ZnONPs complex. (**A**–**C**) Circular dichroism spectra of Hb and <50, 50–100, >100 nm ZnONPs with Hb (**D**–**F**) Time resolved fluorescence spectra of Hb and <50, 50–100, >100 nm ZnONPs with Hb.

CD spectrum of Hb in the presence and absence of ZnONPs was performed at far UV region (200–260 nm) which corresponds to the peptide bond absorption and it can give the information about the helixcity of secondary structure. In the presence of ZnONPs there is a clear systematic effect of the <50, 50–100 and >100 nm ZnONPs on Hb (Fig. 7A–C). Where the intensity of peak is increasing compared to free Hb while decreasing the size of ZnONPs, it indicates the secondary structural changes of protein. The secondary structural components are given in supplementary information Table S3.

### Life time fluorescence spectrum

The time resolved fluorescence decay curve is shown in Fig. 7D–F and decay curve was fitted well to triple-exponential function. Table S4 shows the life time (τ_1_, τ_2_, τ_3_) and relative contribution (A_1_, A_2_, A_3_) of Hb alone and in the presence of various size ZnONPs. The average life time of the fluorescence decay was calculated^27^. The average life time value of Hb is 4.4 which was reduced substantially to1.6, 2.8, and 3.4 with <50, 50–100 and >100 nm ZnONPs. This result indicates the proximity and quenching effect of different sizes of ZnONPs, increases with decreasing size.

## Discussion

Drug induced hemolysis forces the patient to exercise decreased dose of medicine. To date, researchers focus on developing various approaches for designing drugs with high therapeutic potential with minimum side effects. Nanomedicine, especially metal oxide nanoparticles play a significant role in chemotherapy. Among these metal oxide nanoparticles, ZnONPs has attracted more attention for its anticancer efficacy. Despite the rapid progress, several studies report on the toxicity of ZnONPs. Earlier reports showed that the numerous physical characters such as size, shape and stability of nanoparticles are key players to instigate toxicity.

Broad sized NPS showed widened biological effect and on the other hand narrow size ranges showed adverse funnelled biological effect. Density gradient centrifugation allows post synthesis separation of particles according to their size, shape and density^28, 29^. In the present study, ZnONPs of different size range (original size 0–307 nm) are separated by density gradient centrifugation, the three groups of ZnONPs size ranging <50, 50–100 and >100 nm were obtained and its stability was evaluated. The different size was confirmed by DLS and TEM analysis. Systematic and accurate evaluation of stability, solubility and re-dispersity of nanoparticles are a key step before introducing the nanoparticle to biological system. Nanoparticles has tendency to interact with each other or with components present in solvents, used for biological application. These interactions lead to aggregation, changes physical and chemical properties of NPs^30^. Hence the stability of ZnONPs in water, saline, PBS, and FA solution at different storage conditions was assessed and found be increased in order as PBS <saline <FA solution <water. The stability of ZnONPs in water is due to Derjaguin-Landau-Verwey-Overbeek (DLVO) theory. According to DLVO theory, aggregation of nanoparticle depends on the sum of Van der Waals attraction and electric double layer repulsion (EDL)^30^. In case of water EDL is higher which leads to repulsion of nanoparticles and uniform dispersion, which make them stable in water. Whereas in saline and PBS the ionic strength of solvent was increased because of chloride and phosphate ions thus decreasing EDL repulsive energy resulting aggregation^31, 32^. The stability of nanoparticles also depends on storage temperature; the result shows that ZnONPs is most stable in low temperature, in suspended solution nanoparticles sediment because of diffusion force and gravitational force^33^. At higher temperature particle has higher thermal motion which leads to it aggregation and fast sedimentation.

Chicken erythrocyte were used as a model to scrutinize the size depended intake and effect of ZnONPs and ZnONPs conjugate with FA, on structural damage, hemolytic potential, cell viability, ROS generation, and DNA damage. Unlike those of mammals, presence of intact but transcriptionally inactive nucleus and other cell organelles are the most persuasive nature of mature chicken erythrocyte^34, 35^. Hence they are considered as animal cell model to monitor the response to the drug^36, 37^.

The size-dependent hemolytic effect of ZnONPs (>100 nm) on RBCs was assessed, in different solvents and FA, previous reports on the stabilizing effect of FA corroborated our present findings^38, 39^. The hemolytic activity was lesser in presence of FA compared to PBS. The amount of anions presents in PBS significantly affects the stability of ZnONPs. Earlier reports shows that the phosphate ions present in the PBS suppress the hole transfer and decreases the production of ${{\rm{OH}}}^{\bullet }$ radicals in ZnONPs, on the other hand Zn ions release is higher. The higher concentration of metal ions may be the possible reason for increased hemolysis of RBCs observed in present study^32^. However, the size depended impact of ZnONPs on RBCs were observed in case of ZnONPs dispersed in water. In order to check whether the size depended effect is due to ZnONPs or due to soluble Zn ions, we have compared solubility/stability, in terms of Zn ions released into the medium of ZnONPs and ZnCl_2_ (Table S1). The result indicated that Zn ions released from ZnONPs dispersed in water is far away from ZnCl_2_ solution. So we conclude that the impact of ZnONPs on RBC cell is due to the ZnONPs as such. In the presence of FA, hemolysis was lower than water, which might be due to the free radical scavenging property of FA. ZnONPs found to be more stable and less toxic in water and in the presence of FA. Hence, further studies were planned using these vehicles to elucidate toxicity mechanism and its interaction with Hb. The results obtained suggest that the effect of ZnONPs increased in time and concentration depended manner in the presence of water, further ZnONPs (<50 nm) showed greater hemolytic effect compared to larger sizes used in the present study. Several studies corroborate our findings that smaller size nanoparticles show higher toxicity compared to larger particles, as small size nanoparticle easily cross the membrane because of higher surface area and it can strongly interact with biomolecule^40^. The spectacular finding is that the hemolytic effect of ZnONPs is less in presence of FA, may be due its antioxidant effect which protects the cells.

Further we visualized the morphological variations of RBCs treated with ZnONPs in water and in the presence of FA and the result correlated with previous report of RBCs treated with platinum nanoparticles^37^. The <50 nm ZnONPs dispersed in water, treated cells shows elliptocytic RBCs. Iron deficiency leads to development of elliptocytic RBCs, which is common in thalasemia condition. Presence of elliptocytic RBCs in ZnONPs-treated condition infers that the interaction of ZnONPs with Hb and makes the conformational change of Hb and it releases iron from RBCs^41^. The morphological variations are comparatively less in presence of FA as confirmed by SEM. Additionally the viability of RBCs is examined by AO/EtBr staining and LDH activity. The interesting features of chicken RBCs is that the presence of nucleus containing genetic material. AO is taken up by both viable and non-viable cells and emit green fluorescence while intercalating with double stranded DNA and red fluorescence when intercalating with RNA. EtBr is a DNA intercalator, which will be taken up only by non-viable cells and emit red fluorescence^42^. The RBCs treated with <50 nm ZnONPs in water medium contains more non-viable cell compared to 50–100 and >100 nm. Further the uptake of ZnONPs by RBCs was confirmed by ICP-AES and TEM. The emission intensity of ICP-AES is directly proportional to the concentration of elements present (Zn ions) in the RBCs and it was confirmed by TEM photographic image. Significant internalization of <50 nm ZnONPs has been clearly shown in the above data. Among the three different sizes, <50 nm at 50 µg/ml showed statistically significant haemolytic activity (at 24 hr 20% hemolysis), cell internalization and cell death (By AO/EB staining). So we intended to observe the impact of <50 nm size of ZnONPs (50 µg/ml) on DNA and ROS generation. The result indicates that small size ZnONPs permeable through cell membranes damage the DNA through ROS generation or directly interacts with DNA leading to cell death^7^. On the other hand, ZnONPs with FA cause minimal DNA damage. Previous study reported that the interaction of ZnONPs with caffeic acid, which is a precursor of FA^43^, in presence of FA could results in the formation of ZnONPs-ferulate conjugate through the interaction with FA which alleviate its affinity to DNA and protect the cell from DNA damage and cell death. Raman spectra carried out to ascertain the effect of ZnONPs and ZnONPs with FA. Previous report has shown that the Raman spectrum of RBCs is divided into four regions, the first region is low- wave number region, (600–1200 cm^−1^) these region assigned to the symmetric pyrrole deformation and pyrrole breathing mode, the second region is 1200–1300 cm^−1^ which indicates the methane deformation region, third region is pyrrole ring stretching region 1300–1400 cm^−1^ and the last one is spin state marker band region 1500–1650 cm^−1^ ^44^. This result clearly showed the adhesion of <50 nm ZnONPs on Hb. Furthermore the protective effect of albumin on ZnONPs-induced hemolysis was studied. Albumin is the major transport protein present in circulation which has a key role in transport of various types of drugs. The result shows that the protective effect of albumin may be due to high affinity of ZnONPs for albumin. Studies have been shown that ZnONPs interact with albumin to form albumin-ZnO complex which changes the conformation of albumin^45, 46^. To mimic the realistic condition, the effect of ZnONPs and ZnONPs with FA in presence of different percent of serum was studied. Serum starvation is a normal cell surviving process which can induce stress in cancer cell at the same time it protects normal cell from proliferation^47^. The result indicates that hemolytic effect of ZnONPs was increased in serum starved condition but the presence of FA attenuating the hemolytic effect.

Raman spectra of RBCs show the impact of ZnONPs on Hb, so the size-dependent effect of ZnONPs on Hb was also studied. First we studied the UV-visible spectrum of Hb in the presence of <50, 50–100 and >100 nm ZnONPs. The Hb alone shows absorption peaks near UV and visible region, (274 nm) which is known as aromatic region, it indicates the absorption of aromatic amino acid side chain of tryptophan and tyrosine. One sharp intense peak at 414 nm named as soret band or B band and weak intense peaks at 540 and 575 nm is Q bands these are common band in porphyrin compounds, it is due to the transition of four orbitals (two $\pi$ and $\pi$* orbitals) within the heme group enclosed in hydrophobic core of Hb backbone. Soret or B band is due to the transition from ground state to second excited state (S_0_ − S_2_) and Q band is weak transition of electrons to first excited state (S_0_ − S_1_)^48^. The absorption band in the presence of ZnONPs shows significant increase in absorption at 274 nm with slight change in wavelength (hyperchromic red shift) which indicates the change in microenvironment of aromatic amino acid due to the formation of ground state complex with ZnONPs. In case of soret band, slight increase with increasing concentration of ZnONPs which designates the disturbance in structure of Hb. Increase in the intensity of Q band reveals the protonation of Hb porphyrin ring with ZnONPs. The <50 nm ZnONPs were shown more effect when compared to 50–100 and >100 nm ZnONPs. Previous studies have shown that smaller size nanoparticle can easily diffuse into the protein and make changes in protein conformation than larger surface curvature nanoparticle^49^.

Fluorescence emission spectrum reveals the intermolecular distance of fluorophores and interacting molecule, degree of exposure of fluorophores to the solvent and molecular environment of fluorophore. The aromatic amino acids such as tryptophan, tyrosin and phenylalanine are the main intrinsic flurophores present in protein. Depending on the quantum yield, among these amino acids tryptophan is the significant contributor of fluorescence in protein, phenylalanine has poor fluorescent signal^50, 51^. Interactions of proteins with nanoparticles may lead to decrease of fluorescence and is termed as quenching, which is due to the ground state complex formation, excited state reaction, energy transfer and molecular rearrangement. In native folded Hb, tryptophan and tyrosin are located in the hydrophobic pocket and are found to have high quantum yield due to hydrophobic environment. The interaction of Hb with ZnONPs leads to conformational changes in protein which leads to exposure of tryptophan and tyrosine molecule to outside hydrophilic environment leading to decrease of the quantum yield and fluorescence intensity. This phenomenon indicates that ZnONPs can change the conformation and molecular environment of Hb. SFS has relevant roles on interaction studies to characterize the molecular environment around the chromophore present in proteins, this result also supporting the above findings. The conformational changes of secondary structure of Hb were studied by CD spectroscopy. The peak at 209 indicates the π − π* transition of α- helix at 222 is due to π − π* transition of both α- helix and random coil. Where the intensity of peak is increasing compared to free Hb while decreasing the size of ZnONPs, it indicates the secondary structural changes of protein. It is clear that lower size (<50 nm) have high impact on change in helixcity and some degree of unfolding on Hb^52^. While increasing the size (50–100 and >100 nm) the impact of unfolding is very less. This result is good agreement with UV- visible and steady state fluorescence spectroscopy. Time resolved fluorescence spectroscopy were carried out to monitor the fluorescence evolution from Hb as a function of time in the presence and absence of ZnONPs at 280 nm. It helps to investigate the chemical environment and molecular events occurring around the flurophore such as resonance energy transfer, rotational diffusion and macromolecular conformational changes^53^. Fluorescence life time is an average life time of a molecule to remain in excited state before emitting a photon. From the above results it is concluded that <50 nm ZnONPs has higher affinity towards Hb.

## Conclusion

ZnO NPs are considered as one of the promising nanoparticles which could potentially serve as anticancer and drug delivery agent. Biological effect of NPs primarily depends on its stability in different physiological medium. The present study shows that FA could serve as excellent stabilizing agent. Further, our study could prove vital risks associated with different sizes of ZnONPs. The degree of toxicity was found to be enhanced with decrease in the size of NPs. Interestingly; the adverse effect of ZnONPs is minimized in the presence of serum proteins. Similarly FA could prevent ZnONPs induced hemolysis. This novel nano - phytochemical combination would suggest a simple yet efficient approach for biomedical utilization of ZnONPs with reduced side effects. Altogether, our investigation opens up debate on use of ZnONPs alone in biological application, on the other hand results of this study recommends a combination of phytochemicals as desirable method of utilization for various biomedical applications.

## Materials and Methods

### Materials

ZnONPs was purchased from Sigma aldrich (cat. no.677450 and 544906) and FA (C_10_H_10_0_4_, 98%) was obtained from Sisco research laboratories Pvt Ltd. RPMI 1640, MTT, BSA, acridine orange and ethidium bromide were obtained from Himedia.

### Density gradient size separation of ZnONPs

Standard method was adopted to separate the ZnONPs based on its size (Details were given in supplementary information).

### Stability of ZnONPs

Stability of ZnONPs in distilled water, 50 µM FA solution, PBS and saline were analyzed by monitoring hydrodynamic size of the ZnONPs using a Malvern - Zetasizer instrument equipped with a 4 mW He–Ne laser (k = 632 nm). Stock solution of <50, 50–100, and >100 nm ZnONPs was prepared in 1x PBS, water, 50 μM FA solution, and saline were uniformly dispersed by sonication (10 min). The uniformly dispersed stock solution was separated in to three aliquots and kept in different static temperature (Room temperature (RT), 4 °C and −80 °C) and size were monitored 1 h and 24 h. <50 nm size of ZnONPs zeta potential was analysed through Horiba nano particle size analyser, Model SZ-100.

### Solubility of ZnONPs

Solubility of ZnONPs were analyzed by using standard method and quantified by ICP-AES. (Details were given in supplementary information).

### RBC interaction study

#### Hemolysis assay

RBCs were isolated as explained in supplementary information. The haemolytic effect of ZnONPs (100 nm) dispersed in different solvents such as water, PBS and in the presence of FA, were analyzed.

### Morphological study

Morphology of RBCs was examined by phase contrast microscope (Olympus U-RFLT50 at 400X magnification). The RBCs suspension was incubated with 50 μg ml^−1^ of <50, 50–100, and >100 nm ZnONPs in the presence and absence of FA for 24 h. Then the samples were centrifuged and supernatant was removed; pellet was washed with PBS for removing unbounded ZnONPs and the cell suspension was dropped on glass slide and cover slip was placed and examined under the microscope. The morphology was confirmed by scanning electron microscope (SEM) (Hitachi S 3400 N) (details were given in supplementary information)

### Cell viability assay

#### Acridine orange (AO) and ethidium bromide (EtBr) staining (AO/EtBr)

The viability of RBCs in the presence of ZnONPs and ZnONPs with FA (50 µM) was analyzed by AO/EtBr staining. The RBCs suspension was treated with <50, 50–100, and >100 nm ZnONPs of 50 μg ml^−1^ for 24 h. After incubation the cells were washed with PBS, 10 μl of RBCs suspension was placed on the glass slide and 2 μl of staining solution containing AO (1 mg ml^−1^) and EtBr (5 mg ml^−1^) in 1:1 dilution in PBS solution were added mixed well and cover slip was placed and viability was visualized under fluorescent microscope (Olympus U-RFLT50) at 400X magnification.

#### Lactate dehydrogenase (LDH) assay

Lactate dehydrogenase assay was performed to assess the membrane damaging effect of ZnONPs and ZnONPs with FA on RBC cells using Agappe Kit procured from Agappe diagnostics LTD, Agappe hills, Ernanamkulam, Kerala.

### Inductively coupled plasma atomic emission spectroscopy (ICP-AES)

The internalization of ZnONPs in to the RBCs cells were confirmed by ICP-AES. For this study 50 µg ml^−1^ ZnONPs of various size <50, 50–100, >100 nm were treated with RBCs (2 × 10^6^ ml^−1^) and incubated for 24 h. After incubation samples were centrifuged to 3000 rpm for 5 minutes, and then supernatant was removed, cells were washed twice with PBS. The pellet was taken and digested with concentrated nitric acid and further diluted to 5 ml with 1% nitric acid. RBCs without ZnONPs treatment served as control. Solution containing 5, 10, 20, 40, and 80 µg ml^−1^ ZnONPs was used as standard. The emission intensity was found to be directly proportional to the concentration of Zn ions present in the sample which was determined by ICP-AES.

### Transmission electron microscopy

The uptake of ZnONPs by RBCs was confirmed through TEM (Details were given in supplementary information).

### Comet assay

Comet assay was performed as explained in Suyavaran *et al*.^36^. (Supplementary information).

### ROS generation assay

Generation of ROS in RBCs was detected by 2, 7-dichlorohydroflurescein diacetate (DCFDA), 0.2 ml of RBCs (2 × 10^6^ ml^−1^) solution was incubated with 16 µl of <50 nm ZnONPs at various final concentrations 25, 50, 100, 200, 400 μg ml^−1^ in water and in the presence of FA (50 μM) diluted to 1 ml with PBS. This mixture was incubated for 6 h, then sample was centrifuged at 3000 rpm for 4 min to remove the supernatant. The pellet was washed 2 times with PBS, 4 µl of 5 mM DCFDA was added and made up to 1 ml with PBS, the mixture was incubated at 37 °C for 30 min in dark. After incubation the sample was centrifuged at 3000 rpm for 4 min to remove the supernatant. Again samples were washed with 1x PBS, finally the pellet was lysed with 1 ml of distilled water. The fluorescence was measured by Flurolog-FL3-11 spectro-fluorometer with an excitation 495 nm and emission of 520 nm.

### Hemolytic effect of ZnONPs in presence of albumin and fetal bovine serum

Effect of ZnONPs on RBC in presence of albumin (4.5 g dl^−1^; albumin concentration was used based on the normal level of serum) and FBS was studied. Experimental details were given in supplementary file.

### Raman Spectroscopy

The intra erythrocyte events on RBCs upon the addition of ZnONPs was studied under Raman spectroscopy by NRS 3100 Renishaw spectrometer with 785 nm argon laser power of 30 mW. The samples were prepared by treating RBCs using 50 µg ml^−1^. ZnONPs (<50 nm) in the presence and absence of FA and incubated for 6 h. RBCs without ZnONPs treatment were served as control.

### Hb binding study

#### Isolation of Hb

Hemoglobin was isolated from chicken RBCs by adding diluted PBS buffer (1 in 30 with milli Q water) containing only 5 mM NaCl, then the sample was centrifuged for 10 min in 3500 rpm and Hb containing supernatant was collected. The final concentration of Hb was examined by UV-visible spectrophotometer at 415 nm by employing the molar extinction co efficient at 128 mM^−1^ cm^1^ ^54^.

### Interaction of Hb with ZnONPs by spectroscopy

The UV–visible absorbance spectral studies were conducted on shimadzu 1800 spectrophotometer using 1 cm path length and rectangular quartz cuvette. Intrinsic and synchronous fluorescence of Hb in the presence and absence of ZnONPs was measured by using JY Flurolog- FL3-11 spectroflurometer (NRS 3100). The florescence measurement was taken in quartz cuvette with 1 cm path length, protein excited at 280 nm and emission was recorded within the range of 300–450 nm and for the synchronous fluresence the ∆λ value was kept constant at ∆λ 60 nm and ∆λ20 nm. The conformational variations in the secondary and tertiary structure of Hb on binding of ZnONPs were examined by Jasco J815 circular dichroism (CD) spectropolarimeter. The spectra were recorded with in the wavelength range of 200–260 nm using quartz cell of 1.0 cm path length with a scan speed of 50 nm min^−1^. Jasco Spectra Manager II software was used to compute the variation in the percentage of different classes of secondary structure of Hb. Time-resolved fluorescence was examined using FL3-11 spectrofluorometer (NRS 3100), using picoseconds-resolved time correlated single photon counting system with a LED lamp excitation source. The excitation wavelength was 295 nm and number of counts gathered in the channel of maximum intensity was 10,000. The results were analyzed using decay analysis software. (Experiment details were given in supplementary information)

### Statistical analysis

Statistical evaluation was determined using one –way analysis of variance (ANOVA) followed by Turkey’s test using SPSS7.5 software, a value of *P* < 0.05 was premeditated as statistically significant. All experiments were performed in duplicates (analytical) and triplicates. The results were expressed as mean ± SD.

## Electronic supplementary material

Supplementary Information
