# Supplementary material for: Size Dependent Uptake and Hemolytic Effect of Zinc Oxide Nanoparticles on Erythrocytes and Biomedical Potential of ZnO-Ferulic acid Conjugates
Source: Sci Rep. 2017 Jun 23;7:4203. doi: 10.1038/s41598-017-04440-y (PMC5482866; doi:10.1038/s41598-017-04440-y)
Supplement: Supplementary file 1 — Supplementary Information [file 41598_2017_4440_MOESM1_ESM.pdf]

Supplementary Information

Size Dependent Uptake and Hemolytic Effect of Zinc Oxide Nanoparticles on Erythrocytes and Biomedical Potential of ZnO-Ferulic acid Conjugates

E. Preedia Babu[a], A. Subastri[a] , A. Suyavaran[a], K. Premkumar[b], V. Sujatha[c], B. Aristatile[d], Ghedeir M. Alshammari[d], V. Dharuman[e] and C. Thirunavukkarasu[a*]

[a]*Department of Biochemistry and Molecular Biology, Pondicherry University, Puducherry 605 014, India.*

[b]*Cancer Genetics and Nanomedicine Laboratory, Department of Biomedical Science, Bharathidasan University, Tiruchirappalli-620 024.*

[c]*Department of Chemistry, Periyar University, Salem-636011, India.*

[d]*Department of Food Science and Nutrition, College of Food and Agricultural Science, King Saud University, P.O. Box 2460, Riyadh 11451, Saudi Arabia.*

[e]*Molecular Electronics Laboratory, Department of Bioelectronics and Biosensors, School of Life Sciences, Alagappa University, Karaikudi - 630 003. India*

** Corresponding author: Tel.:+91 413 2654972 or* +011 347 471 6477*; Fax: +91 413 2655255;*

E-email address: tchinnasamy@hotmail.com or tarasu.bbm@pondiuni.edu.in *(C.Thiru)*

Methods

Density gradient size separation of ZnONPs

Sucrose density gradient were used for ZnONPs size separation, different concentrations of sucrose were prepared (70%, 50%, 25%, 10%) and layered in 8ml polycarbonate ultracentrifuge tube. The tube was layered with 2.5ml 70% sucrose solution followed by 1ml of 50%, 20%, 10% sucrose solution and 0.5ml of ZnONPs. Before adding to sucrose gradient, ZnONPs were uniformly dispersed through sonication for 10 min in 60% pulsation mode and sonication power of 60 W by using Branson digital sonicator. The samples were centrifuged at 1000 rpm for 10 min in ultracentrifuge (Sorvall RC 90). After the centrifugation the gradient was collected and resuspended in water. Further the average hydrodynamic size of ZnONPs present in each gradient layer was analyzed by using Malvern-Zetasizer instrument which was equipped with a 4mW He-Ne laser. Further the size of separated ZnONPs was visualized through Transmission electron microscopy (TEM) (Jeol/JEM2100) with an acceleration voltage of 200KV.

Solubility of ZnONPs

The solubility of ZnONPs (<50nm) in distilled water was analyzed by measuring the concentration of Zn ions using inductively coupled plasma atomic emission spectrometry (ICP-AES) by Perkin Elmer Optima 5300DV. 1mg/ml concentration of <50 nm ZnONPs or $ZnCl_2$ was uniformly dispersed (dissolved in case of $ZnCl_2$) in distilled water by sonication (10 min). The uniformly dispersed solution were made into three aliquots incubated at different static temperature (RT, 4°C and -80°C; $ZnCl_2$ only RT) for 1 h and 24 h. After incubation the solution was centrifuged at 14,000 rpm at 25°C for 6 h in Eppondrof centrifuge 5810R. Further 100-200µl of supernatant was taken and digested with concentrated nitric acid and diluted to 5 ml

with 1% nitric acid. The concentration of Zn ions were measured by ICP-OES. Values are expressed as µg of Zn ions per 100 µg of ZnONP or $ZnCl_2$.

Preparation of red blood cells

Chicken blood sample was used for all the experiments; blood samples were freshly collected from slaughter house near Pondicherry University in EDTA containing 50 ml centrifuge tube. Blood sample was centrifuged at 2500 rpm for 5 min and RBCs were separated from plasma, then RBCs were washed with 1x PBS for 3 times. The purified RBCs were suspended in 1x PBS and the number of RBCs was counted by using hemocytometer.

Hemolytic potential

The Hemolysis inducing effect of ZnONPs (100 nm) dispersed in different solvents such as water, PBS, saline and in presence of FA (50 µM), on RBCs were analyzed. For this assay 16 µl ZnONPs at various final concentrations (25, 50,100,200,400,800,1600 µg ml^{-1}) in the above solvents were added to 0.2 ml of diluted RBCs (2×10^6 ml^{-1}) and made up to 1 ml with PBS. Instead of PBS water was used as positive control and 16 µl of various solvents without ZnONPs incubated with RBCs were served as negative control; the samples were mixed gently and kept in 37 °C for 24 h. After 24 h incubation the mixture was centrifuged at 3000 rpm for 3 min and 100 µl of supernatant was transferred to microwell plate and absorbance read at 570 nm by using ELSA plate reader.

The same hemolytic activity was assessed with different sizes (<50, 50-100 and >100 nm) ZnONPs of concentrations 25, 50, 100, 200, 400, 800 µg ml^{-1} were dispersed in water and with FA (50 µM) at various time intervals (2 h, 4 h, 6 h and 24 h). The hemolysis percent of RBCs was calculated by using the following formula

$$\text{Percent hemolysis of RBCs} = \left(\frac{\text{Samble absorbance} - \text{negative control absorbance}}{\text{positive control absorbance} - \text{negative control absorbance}}\right) \times 100 \quad (1)$$

Scanning electron microscopy (SEM)

The morphological changes of RBCs were visualized after incubation with ZnONPs (50 μg ml^{-1} of <50, 50-100 and >100 nm) for 24 h; after incubation the samples were centrifuged and the supernatant was removed and finally washed with PBS solution. The RBCs cells were diluted to 1: 200 and 200 µl was placed on the cover slip, the cells were fixed by 25% glutaraldehyde and methanol. The fixed sample was analyzed under scanning electron microscope (Hitachi S 3400N).

Visualizing ZnONPs uptake through Transmission electron microscopy

The uptake of ZnONPs by RBC cells were visualized after incubation with ZnONPs (50 μg ml^{-1}) for 24 h. After incubation the cells were collected and fixed in 2.5% glutaraldehyde prepared in 0.1M sodium cacodylate buffer (pH 7.2) for 4 h (4°C). The solution was centrifuged and the cells were washed with the same buffer (3x 10 min) and 0.1% osmium tetroxide (OsO_4) was added. This post fixation was carried out for 2 h at 4°C. The excess fixative was washed with the same buffer (3x 10 min). The RBCs were then treated with graded series of 30%, 50%, 70%, 80%, 90% and 100% acetone, twice for ten minutes each followed by propylene oxide treatment twice with ten minutes each. The sedimented cells were then infiltrated with the resin mixture and propylene oxide, where the resin mixture consisted of Epon 812 resin, DDSA (Dodecenyl succinic Anhydride) and NMA (Nadic® Methyl anhydride) starting with 25%, 50% and 75% for 2 h at each concentration and then finally with 100% resin (2x 2 h). The specimen was finally embedded using the same resin mixture with added catalyst (DMP 30) in “easymoulds” at 60°C for 48 h. The resin blocks were removed from the mould, trimmed and sectioned using Leica

Ultracut R Ultramicrotome with diamond or glass knives. Initially semi thin sections were cut which were stained with toluidine blue and screened using the light microscope to check for area of interest in those sections from that particular block. Then ultrathin sections were cut, collected on copper grids and stained with saturated solutions of uranyl acetate followed by lead citrate. After air drying, the sections were screened in JEOL JEM 1400 TEM at an accelerating voltage of 80 kV. The micrographs were taken using the Olympus keen view CCD Camera attached to the microscope.

Comet assay

Single cell gel electrophoresis or comet assay is a simple method for visualization of damaged DNA. Comet assay was performed, in detail glass slides were coated with 1% normal melting point agarose. The RBCs (2×10^6 ml^{-1}) was treated with 50 µg ml^{-1} ZnONPs (<50 nm) in the presence and absence of FA for 24 h, RBCs without treatment was served as control. The samples (25 µl) were diluted to 200 µl with 0.5% low melting agarose and layered over the agarose coated slide. The slides were kept in lysis buffer for 1 h; then moved to electrophoresis running buffer for 15 min and electrophoresis was done for 30 min at 50 v, then the slides were kept in neutralizing buffer for 15 min, then the slides were stained with EtBr of 10 µg ml^{-1} concentrations and observed under fluorescent microscope.

Hemolytic effect of ZnONPs in presence of albumin and fetal bovine serum

For this study we used hemolytic concentration of ZnONPs 16 µl ZnONPs (200 and 400 µg ml^{-1}) at <50 nm size were added to 0.2 ml of RBCs (2×10^6 ml^{-1}) and made up to 1 ml with PBS in the presence and absence of albumin and was incubated for 24 h, after incubation samples were centrifuged at 3000 rpm for 5 min and supernatant was transferred to microwell plate and the readings were taken at 570 nm and thus, percentage of hemolysis was calculated.

Further ZnONPs (200 µg ml^{-1}) <50 nm size and ZnONPs with ferulic acid (FA) (50 µM) were added to 0.2ml of RBCs (2×10^6 ml^{-1}) in presence of 0% , 3.125%, 6.25%,12.5%. 25 % FBS.The samples were incubated for 24h after incubation samples were centrifuged at 3000 rpm for 5 min and supernatant was transferred to microwell plate and the readings were taken at 570 nm and thus, percentage of hemolysis was calculated.

Hemoglobin Interaction study

Preparation of sample

The stock solutions of <50, 50-100, and >100 nm ZnONPs (10 mg ml^{-1}) were prepared in double distilled water and to have uniform dispersion, the samples were sonicated for 10 min, after sonication 25, 50, 100, 200, 400 µgml^{-1} ZnONPs were used for Hb interaction studies.

Intrinsic fluorescence study

The kinetic efficacy of fluorescent quenching was assessed by Stern- Volmer quenching equation.

$$F_0/F = 1 + Ksv\,[Q] \qquad (2)$$

F_0/F is the ratio of fluorescence intensity of Hb in the absence and presence of ZnONPs, Ksv is the and [Q] is the concentration of ZnONPs. The Ksv value is calculated from Stern – Volmer quenching plot of F_0/F Vs [Q]. Quenching is commonly of two types, static quenching and dynamic quenching, it can be differentiated by bimolecular quenching constant. The type of quenching is calculated from the following equation.

$$Kq = Ksv/\tau_0 \qquad (3)$$

Where, Kq is the bimolecular quenching constant and τ_0 is the life time of quencher in the absence of flurophore. The value of τ_0 is approximately 2.58×10^{-9} s for Hb.

Results

Table. S1. The concentration Zn ions present the dispersed solution of ZnONPs and $ZnCl_2$ at different static temperature. Values are presented as mean± SD (n=3), expressed as mean percentage, # value represent significant difference at $P \leq 0.05$ of $ZnCl_2$ sample.

**SAMPLES**		**1 h**	**24 h**
**ZnONPs (µg of Zn ions /100 µg of ZnONPs) (<50 nm)**	**RT**	**0. 312±0.02#**	**0.491±0.04#**
	**4°C**	**0.148±0.03#**	**0.370±0.03#**
	**-80°C**	**0.128±0.04 #**	**0.268±0.02 #**
**$ZnCl_2$ (µg of Zn ions/ 100µg of $ZnCl_2$)**	**RT**	**44 ±4**	**45 ±6**

Table. S2. The mean percent of DNA present in head and tail of control RBCs, RBCs treated with <50 ZnONPs alone and <50 ZnONPs treated with FA (50µM) was determined by imageJ software. Values are presented as mean± SD (n=3), expressed as mean percentage, # value represent significant difference at *P*≤0.05 of control sample

Samples	Size	% of DNA in head	% of DNA in tail
**Control**		95.5±4	4.5±5
**ZnONPs in water**	<50 nm	15.01±5#	84.99±4#
**ZnONPs with FA (50 µM)**	<50 nm	82.25±3#	20.83±3#

Table S3. Secondary structure components of free hemoglobin and hemoglobin treated with <50, 50-100 and >100 nm ZnONP at various concentrations was assessed using Jasco Spectra Manager II software

Samples	Secondary structure components (%)			
	α helix	β sheet	Turn	Random
**Free hemoglobin**	60.0	0.6	18	21.4
**<50 nm ZnONPs + Hb**				
**25 μg ml^{-1}**	59.2	1.1	18.5	20.9
**50 μg ml^{-1}**	57.5	2.5	18.3	21.7
**100 μg ml^{-1}**	56.4	2.6	17.5	23.8
**200 μg ml^{-1}**	53.6	3.0	16.5	26.9
**50 - 100 nm ZnONPs + Hb**				
**25 μg ml^{-1}**	60	1.2	17.8	21
**50 μg ml^{-1}**	58.8	1.8	17.5	21.9
**100 μg ml^{-1}**	57.5	2.1	17	23.4
**200 μg ml^{-1}**	56.9	2.7	16.9	23.5
**>100 nm ZnONPs + Hb**				
**25 μg ml^{-1}**	61.1	0.4	18.6	19.9
**50 μg ml^{-1}**	60	1.2	18.2	20.6
**100 μg ml^{-1}**	59	1.3	17.8	21.5
**200 μg ml^{-1}**	59.3	1.5	17.5	21.7

Table S4. Fluorescence lifetime data of hemoglobin in presence of 0-50, 50-100, above 100 nm ZnONPs (50µg ml^{-1})

**Samples**	**A_1**	**A_2**	**A_3**	**τ_1**	**τ_2**	**τ_3**	**τav**
**Control**	0.20	0.21	0.58	2.3	5.4	0.017	4.4
**<50 nm**	0.006	0.003	0.99	1.4	5.7	0.043	1.6
**50-100 nm**	0.019	0.006	0.98	1.4	5.3	0.023	2.8
**>100 nm**	0.0544	0.032	0.91	1.3	5.2	0.044	3.4

Figure S1. The ZnONPs was separated by density gradient centrifugation and the size distribution of ZnONPs was determined by DLS analysis and the size distribution was visualized by transmission electron photomicrograph (A) ZnONPs was penetrated through the sucrose gradient and settled as three band at 25%, 50% and 75% sucrose gradient (B - E) DLS analysis of ZnONPs before separation by density gradient, after separation ZnONPs in 25% sucrose gradient (<50 nm), ZnONPs in 50% sucrose gradient (50 - 100 nm), ZnONPs in 75% sucrose gradient (>100 nm). (F-I) TEM images showing size of ZnONPs (F-before separation; G-<50nm; H- 50-100 nm & I- >100 nm).

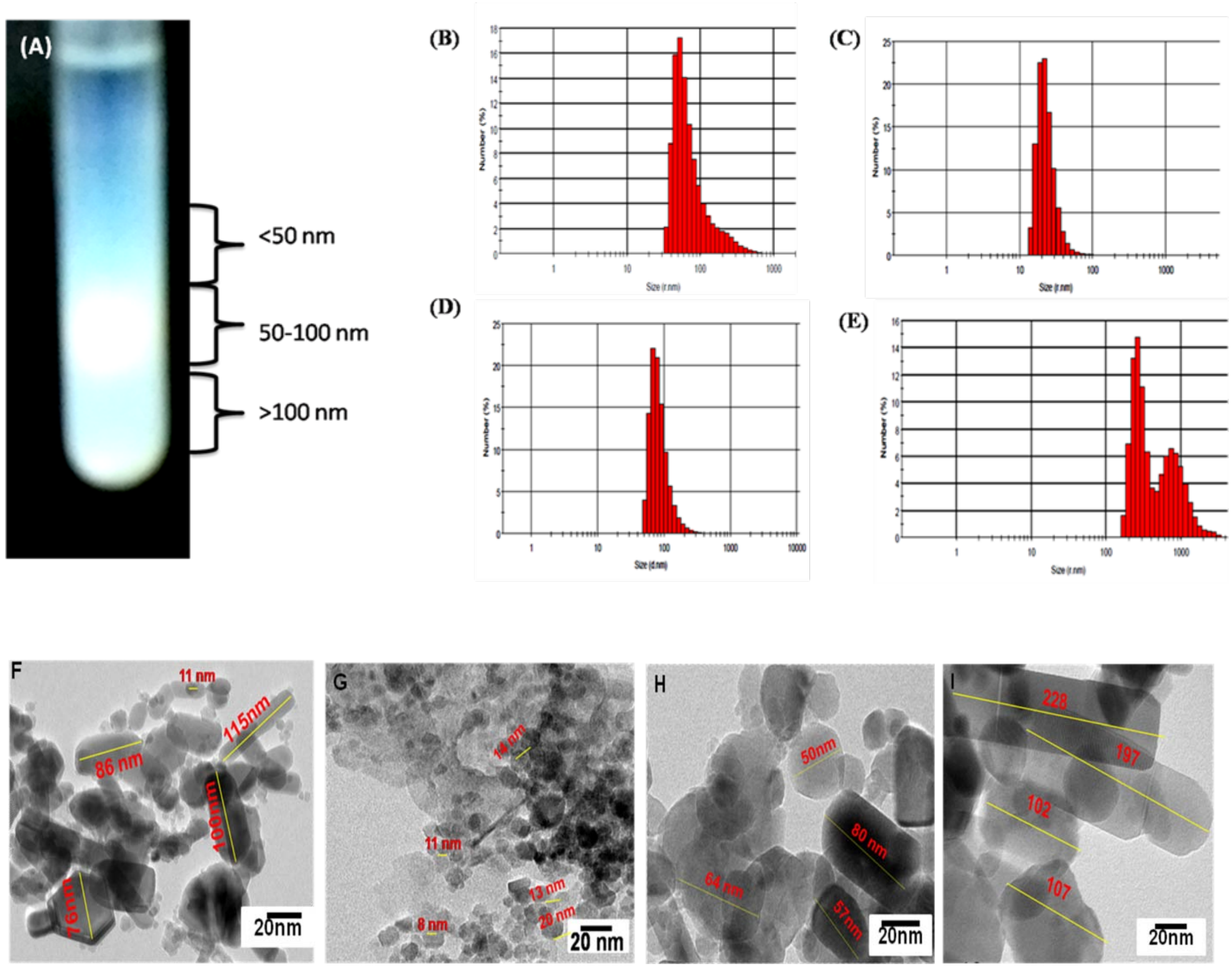

Figure S2. The stability of ZnONPs was analyzed by Zeta potential analysis. The stability of ZnONPs in different solvent was analyzed by zeta potential analysis (A) in water (B) FA solution (C) PBS (D) Saline

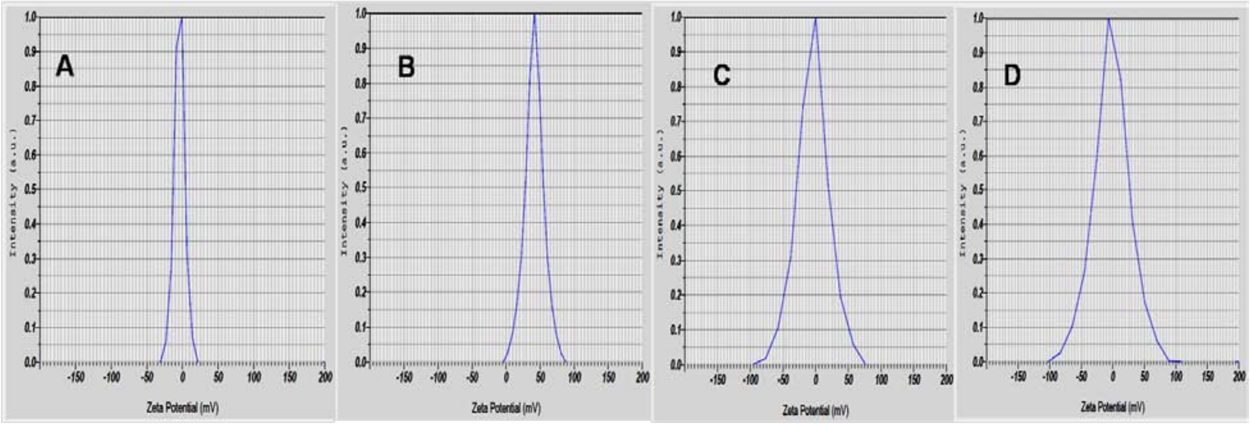

Figure S3. Effect of ZnONPs and ZnONPs with FA on human RBCs: The percent hemolysis of human RBCs incubated (24 h) with <50 nm ZnONPs and ZnONPs with FA.

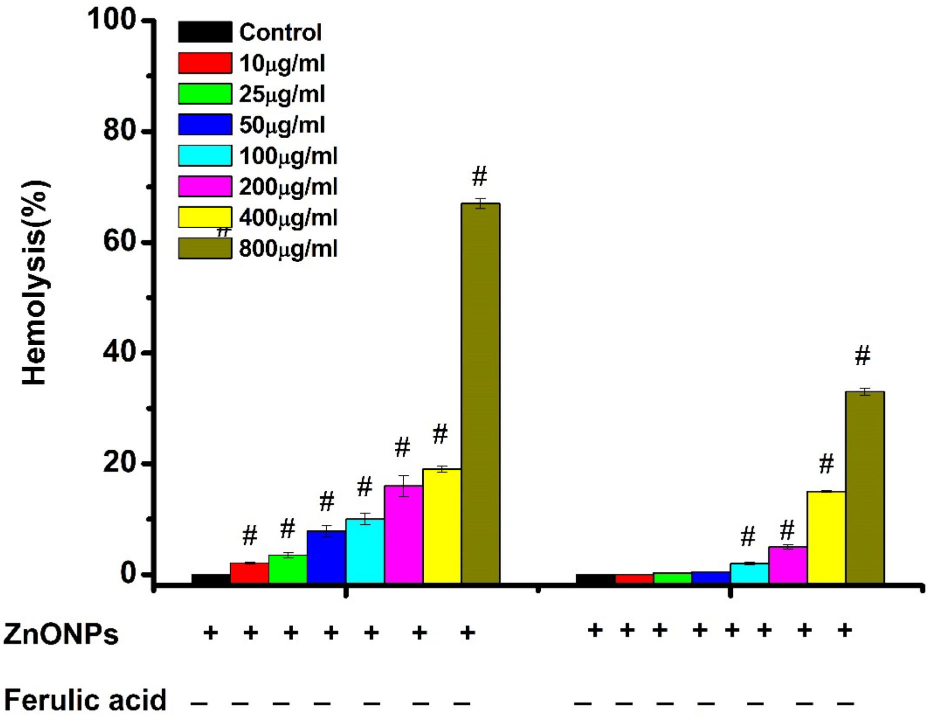

Figure S4. Effect of ZnONPs and ZnONPs with FA on membrane integrity: The release of LDH due to the effect of ZnONPs (100, 200, 400μg ml^{-1}) and ZnONPs with FA on erythrocytes membrane.

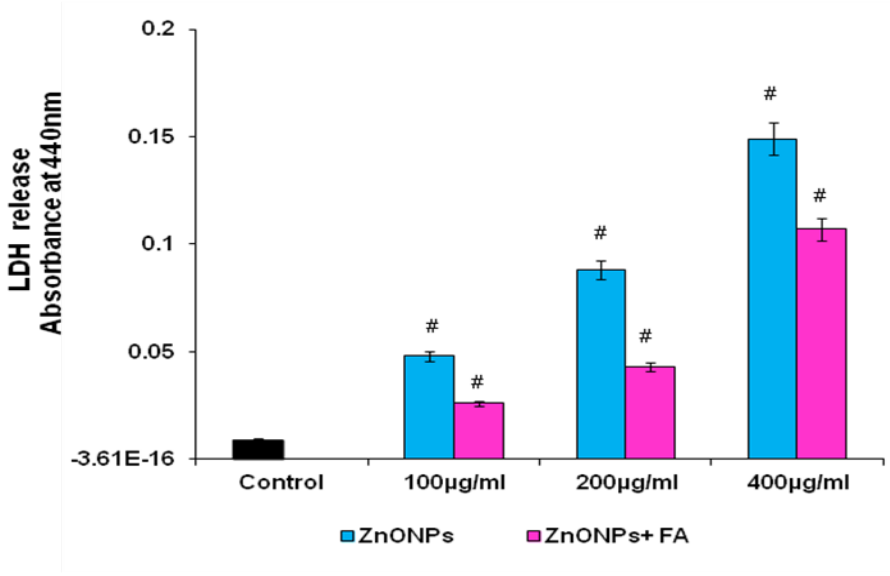

Figure S5. Photograph of hemolysis induced by <50 nm ZnONPs in presence and absence of albumin. (1) 200 μg ml^{-1} ZnONPs + Albumin (4.5 μg dl^{-1}), (2) 400 μg ml^{-1} ZnONPs + Albumin (4.5 μg dl^{-1}) , (3) Normal RBCs in presence of albumin,(4) RBCs alone, (5) 100 μg ml^{-1} ZnONPs , (6) 200 μg ml^{-1} ZnONPs.

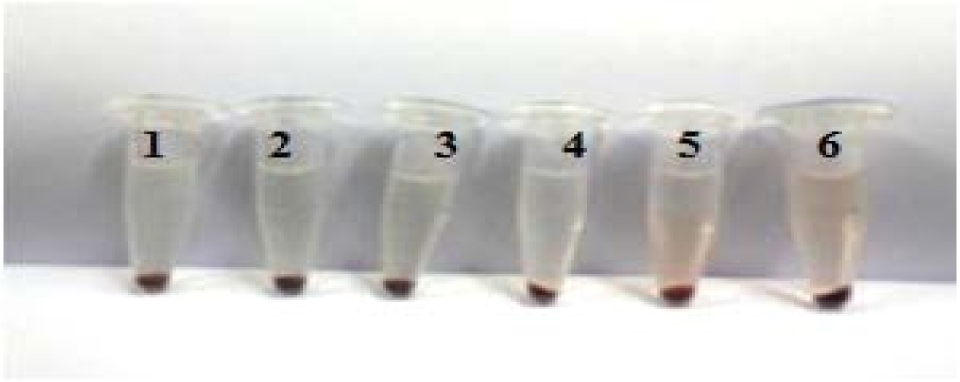

Figure S6. Efficiency of fluoresence quenching of Hb. Stern Volmer plot of Hb Fluorescence quenching by (A-C) <50 nm ZnONPs , 50-100 nm ZnONPs, >100 nm ZnONPs.

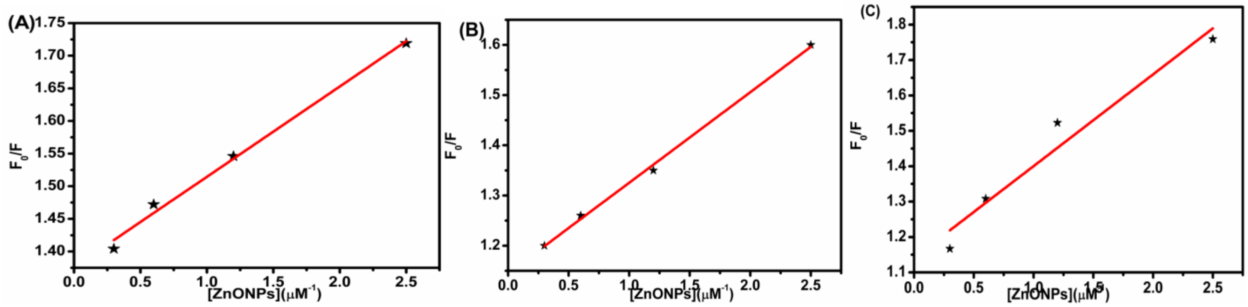

Figure S7. Molecular environmental changes of hemoglobin tyrosin residue with various size and concentration of ZnONPs were analyzed by synchronous fluorescence spectroscopy. (A-C) Synchronous fluorescence (Δλ = 20 nm) spectra of <50, 50-100, >100 nm Hb – ZnONPs

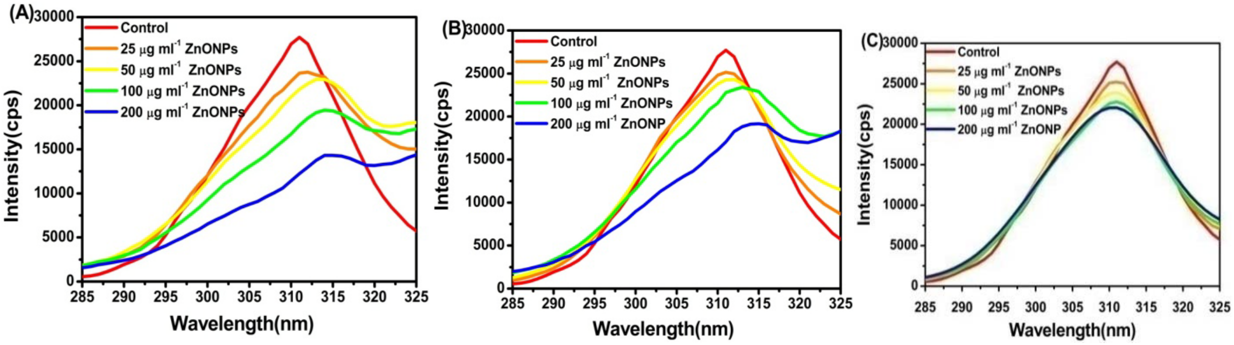

.
